# Augmented Chondroitin Sulfate Proteoglycan Has Therapeutic Potential for Intervertebral Disc Degeneration by Stimulating Anabolic Turnover in Bovine Nucleus Pulposus Cells under Changes in Hydrostatic Pressure

**DOI:** 10.3390/ijms22116015

**Published:** 2021-06-02

**Authors:** Yoshiki Takeoka, Phani Paladugu, James D. Kang, Shuichi Mizuno

**Affiliations:** Department of Orthopaedic Surgery, Brigham and Women’s Hospital and Harvard Medical School, 75 Francis Street, Boston, MA 02115, USA; ytakeoka@bwh.harvard.edu (Y.T.); phani_paladugu@hms.harvard.edu (P.P.); jdkang@bwh.harvard.edu (J.D.K.)

**Keywords:** intervertebral disc degeneration, in vitro nucleus pulposus tissue model, cyclic and constant hydrostatic pressure, osmolality and osmotic pressure, biomaterial, chondroitin sulfate proteoglycan, hyaluronan, anabolic turnover, TRPV4, spine

## Abstract

Nucleus pulposus (NP) cells are exposed to changes in hydrostatic pressure (HP) and osmotic pressure within the intervertebral disc. We focused on main disc matrix components, chondroitin sulfate proteoglycan (CSPG) and hyaluronan (HA) to elucidate the capability of augmented CSPG to enhance the anabolism of bovine NP (bNP) cells under repetitive changes in HP at high osmolality. *Aggrecan* expression with CSPG in the absence of HP was significantly upregulated compared to the no-material control (phosphate buffer saline) under no HP at 3 days, and *aggrecan* expression with CSPG under HP was significantly higher than the control with HA under HP at 12 days. *Collagen type I* expression under no HP was significantly lower with CSPG than in controls at 3 days. Although *matrix metalloproteinase 13* expression under HP was downregulated compared to no HP, it was significantly greater with HA than the control and CSPG, even under HP. Immunohistology revealed the involvement of mechanoreceptor of transient receptor potential vanilloid-4 activation under HP, suggesting an HP transduction mechanism. Addition of CSPG had anabolic and anti-fibrotic effects on bNP cells during the early culture period under no HP; furthermore, it showed synergy with dynamic HP to increase bNP-cell anabolism at later time points.

## 1. Introduction

Back pain is a global health problem with a considerable socioeconomic burden, and intervertebral disc (IVD) degeneration is one of the major independent risk factors [1]. However, current surgical treatments for IVD degenerative diseases, e.g., pathological disc excision and/or spinal fusion, result in the loss of some spinal function. Thus, the development of regenerative therapy has been pursued; however, its success has been met with such obstacles as avascularity in IVD, lack of regenerative capability of resident cells, and incessant mechanical loading. The IVD has a complex structure, with the amorphous nucleus pulposus (NP) confined by the collagenous annulus fibrosus and cartilaginous endplates, supporting compressive loading and facilitating multidimensional spinal movement [2]. Furthermore, the IVD is not only immune-privileged, but is also the largest avascular organ in the body [3], which places resident cells in an extremely harsh environment—low glucose, oxygen, and pH and high osmotic pressure (OP) and load repetition [4].

To develop therapeutic strategies for IVD diseases, we have been focusing on the physiological microenvironment and homeostasis within NP. The NP contains highly negatively charged extracellular matrix (ECM), which is capable of absorbing abundant interstitial fluid [3,5] and generating high OP [6,7]. The NP is also exposed to changes in hydrostatic pressure (HP) in daily cycles owing to weight bearing in the upright position and off-loading in the recumbent position [8,9]. Our latest studies demonstrate that a repetitive regimen of cyclic HP followed by constant HP at high osmolality stimulated anabolic gene upregulation and dense accumulation of ECM in bovine NP (bNP) cells [10,11]. Therefore, the combination of dynamic HP and intradiscal high OP is required in maintaining bNP-cell homeostasis [10,12].

In addition to the aforementioned cellular responses to changes in HP at high OP, approaches using biomaterials and exploiting endogenous cell populations to restore cellular properties and stimulate ECM production have gained increased attention in the field of IVD regeneration [13]. While no biomaterials are clinically approved for IVD repair, mainly due to safety concerns and unclarified repair mechanisms, we selected chondroitin sulfate proteoglycan (CSPG) and hyaluronan (HA) as candidate therapeutic materials, as they are the main components of disc and cartilage ECM. Both CSPG and HA attract interstitial water, contributing to the microenvironment and mechanical structure of the NP [14]. Aggrecan, the primary CSPG in the IVD, is characterized by its highly negative charge density, owing to sulfate chains [15,16]. The HA is a unique, non-sulfated glycosaminoglycan whose molecular weight reaches the millions [17] and has both anti-inflammatory and anabolic effects in the IVD [18]. The concept behind our novel material-based therapy is to implant extracted CSPG into degenerated NP, which prevents progressive degeneration and ultimately promotes regeneration. Based on our previous studies [10,11], we hypothesized that augmentation of CSPG stimulates the anabolic capability of bNP cells under repetitive changes in HP at high osmolality. To test that hypothesis, we incubated isolated bNP cells with CSPG or HA under a repetitive regimen of cyclic HP at 0.2–0.7 MPa, 0.5 Hz for 2 days, followed by constant HP at 0.3 MPa for 1 day at high osmolality (450 mOsm/kg H_2_O) for up to 12 days, and compared the gene expression and immunohistology of metabolic markers in the bNP cells. We also sought to clarify the involvement of mechanoreceptor of transient receptor potential vanilloid-4 (TRPV4) activation in bNP-cell metabolism [19,20].

## 2. Results

### 2.1. The Effects of Augmenting ECM on Metabolism in bNP Cells under HP

We compared the expression of ECM-related genes in bNP cells augmented with CSPG or HA and incubated under HP loading or no HP. We also assessed the histological characteristics of accumulated keratan sulfate (KS) as the specific glycosaminoglycan chain of aggrecan to support the gene expression of *aggrecan* core protein (*Acan*).

**Figure 1:**
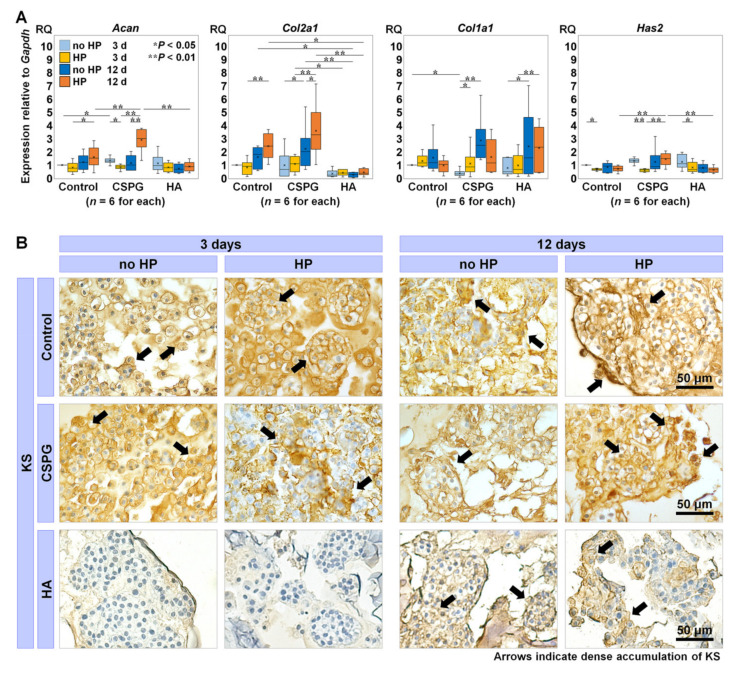
The effects of augmenting extracellular matrix (ECM) on metabolism in bovine nucleus pulposus (bNP) cells under hydrostatic pressure (HP). (**A**) Gene expression of *aggrecan* core protein (*Acan*), *collagen type II* and *I* (*Col2a1* and *Col1a1*), *hyaluronan synthase 2* (*Has2*) with the no-material control, chondroitin sulfate proteoglycan (CSPG), and hyaluronan (HA) under no HP and HP at 3 and 12 days relative to the control under no HP at 3 days (*n* = 6 for each). Data are presented with box plots. Multi-way repeated measures analysis of variance (ANOVA) with the Bonferroni post hoc test was used. * *p* < 0.05 and ** *p* < 0.01. *Gapdh*, *glyceraldehyde 3-phosphate dehydrogenase*. RQ, relative quantity. (**B**) The accumulation of keratan sulfate (KS) in brown counterstained with hematoxylin. Arrows indicate intense accumulation, each section is 7 μm thick, and the bar indicates 50 μm.

The gene expression of *Acan* in bNP cells with CSPG under no HP was significantly upregulated compared to the no-material control under no HP at 3 days (*p* = 0.03). Under HP, *Acan* expression with CSPG was significantly higher than the control *(p* = 0.007) at 12 days. In addition, *Acan* expression with CSPG at 12 days was also significantly greater under HP than no HP (*p* < 0.001). On the other hand, *Acan* expression with HA (augmented as another main ECM component) under HP was significantly downregulated compared to CSPG under HP at 12 days (*p* < 0.001) (Figure 1A). The expression of *collagen type II* (*Col2a1*) in bNP cells with CSPG under HP was significantly upregulated compared to no HP at 12 days (*p* = 0.02). With HA, however, *Col2a1* expression was significantly lower than in the no-material control, with or without HP, at 12 days (*p* = 0.01 and *p* = 0.02, respectively) (Figure 1A). The expression of *collagen type I* (*Col1a1*) in bNP cells with CSPG under no HP was significantly downregulated compared to the no-material control under no HP at 3 days (*p* = 0.02). With HA under HP, *Col1a1* expression was significantly higher at 12 days than 3 days (*p* = 0.006) (Figure 1A). The expression of *hyaluronan synthase 2* (*Has2*) in bNP cells with CSPG under HP was significantly upregulated compared to the no-material control under HP at 12 days (*p* = 0.002). The *Has2* expression with HA under HP was significantly lower than that of CSPG under HP at 12 days (*p* = 0.001) (Figure 1A).

Immunohistological staining showed KS accumulation within the bNP cells/clusters in the no-material control and CSPG, in which accumulation was particularly dense at 12 days. Meanwhile, relatively little accumulation of KS was found in the bNP cells/clusters with HA, which was denser at 12 days than at 3 days (Figure 1B).

These results indicate that augmenting CSPG enhances anabolic turnover and suppresses gene expression of fibrotic molecules in bNP cells at early time points. In addition, CSPG is suggested to have a synergistic effect with dynamic HP on ECM synthesis at later time points (12 days). However, augmenting HA does not appear as beneficial as CSPG for anabolic turnover in bNP cells.

### 2.2. The Effects of Augmenting ECM on Catabolic Turnover in bNP Cells under HP

We compared the expression of catabolic and anti-catabolic genes in bNP cells augmented with CSPG or HA, as well as between the loading conditions. We also performed immunohistological staining against matrix metalloproteinase 13 (MMP13).

**Figure 2:**
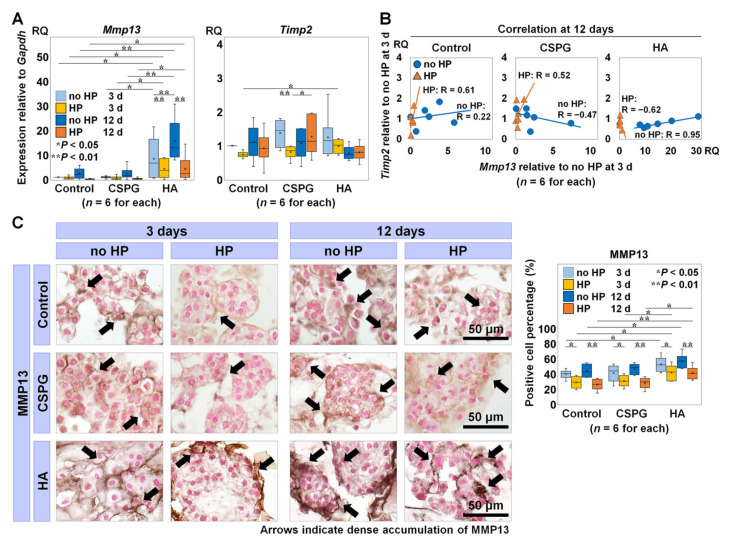
The effects of augmenting extracellular matrix (ECM) on catabolic turnover in bovine nucleus pulposus (bNP) cells under hydrostatic pressure (HP). (**A**) Gene expressions of catabolic *matrix metalloproteinase 13* (*Mmp13*) and anti-catabolic *tissue inhibitor of metalloproteinases 2* (*Timp2*) with the no-material control, chondroitin sulfate proteoglycan (CSPG), and hyaluronan (HA) under no HP and HP at 3 and 12 days relative to the control under no HP at 3 days (*n* = 6 for each). Data are presented with box plots. Multi-way repeated measures analysis of variance (ANOVA) with the Bonferroni post hoc test was used. * *p* < 0.05 and ** *p* < 0.01. *Gapdh*, *glyceraldehyde 3-phosphate dehydrogenase*. RQ, relative quantity. (**B**) The correlation between *Mmp13* and *Timp2* expression with the control, CSPG, and HA under no HP and HP at 12 days relative to the control under no HP at 3 days (*n* = 6 for each). Pearson correlation analysis was used. (**C**) The accumulation of MMP13 in black counterstained with Contrast Red and positive cell percentage of MMP13 (*n* = 6 for each). Arrows indicate intense accumulation, each section is 7 μm thick, and the bar indicates 50 μm. Data are presented with box plots. Multi-way repeated measures ANOVA with the Bonferroni post hoc test was used. * *p* < 0.05 and ** *p* < 0.01.

The gene expression of *Mmp13* in bNP cells was downregulated under HP compared to no HP in the control, with CSPG, and with HA both at 3 and 12 days, although only HA produced a statistically significant difference (3 days, *p* = 0.004; 12 days, *p* < 0.001). The *Mmp13* expression with HA was significantly upregulated compared to the no-material control regardless of HP at each time point (no HP at 3 days, *p* = 0.02; HP at 3 days, *p* = 0.02; no HP at 12 days, *p* = 0.001; HP at 12 days, *p* = 0.03) and compared to CSPG (no HP at 3 days, *p* = 0.02; HP at 3 days, *p* = 0.02; no HP at 12 days, *p* = 0.001; HP at 12 days, *p* = 0.04) (Figure 2A). The expression of *tissue inhibitor of metalloproteinases 2* (*Timp2*) in bNP cells with CSPG under HP was significantly higher at 12 days than at 3 days (*p =* 0.01) (Figure 2A).

We performed a Pearson analysis to assess the correlation between *Mmp13* and *Timp2* gene expression with each material at 12 days. The value in the no-material control under no HP at 3 days was set as 1.0. The scatter plot demonstrated *Mmp13* suppression under HP compared to no HP with each material, while *Timp2* expression did not differ between no HP and HP. In bNP cells with the control and CSPG, positive correlations were found between *Mmp13* and *Timp2* under HP at 12 days (*R* = 0.61 and *R* = 0.52, respectively) (Figure 2B).

Immunohistology showed MMP13 to be denser within the bNP cells/clusters under no HP than HP in each material. In addition, bNP cells/clusters with HA showed denser MMP13 than the no-material control and CSPG regardless of HP at each time point. The percentage of cells positive for MMP13 was significantly higher under no HP than HP with all materials, both at 3 and 12 days (control, 3 days, *p* = 0.03; 12 days, *p* = 0.001; CSPG, 3 days, *p* = 0.03; 12 days, *p* = 0.001; HA, 3 days, *p* = 0.03; 12 days, *p* = 0.003), and significantly higher with HA than the control regardless of HP (no HP at 3 days, *p* = 0.03; HP at 3 days, *p* = 0.02; no HP at 12 days, *p* = 0.02; HP at 12 days, *p* = 0.004) and then CSPG under HP (3 days, *p* = 0.04; 12 days, *p* = 0.01) (Figure 2C).

These results demonstrate that repetitive changes in HP elicit catabolic *Mmp13* downregulation in the no-material control, with CSPG, and with HA and anti-catabolic *Timp2* upregulation in response to slight *Mmp13* upregulation with the no-material control and CSPG. Conversely, HA augmentation promotes catabolic turnover regardless of the presence of HP.

### 2.3. Involvement of TRPV4 in the Effects of Augmenting ECM on bNP Cells under HP

The bNP cells were exposed to cyclic and constant HP applied using our hydrostatic pressure culture system. We stained bNP cells/clusters immunohistologically against TRPV4, a mechanosensitive calcium-permeable channel [19,20], in an attempt to clarify the possible mechanisms of HP effects on bNP cells.

**Figure 3:**
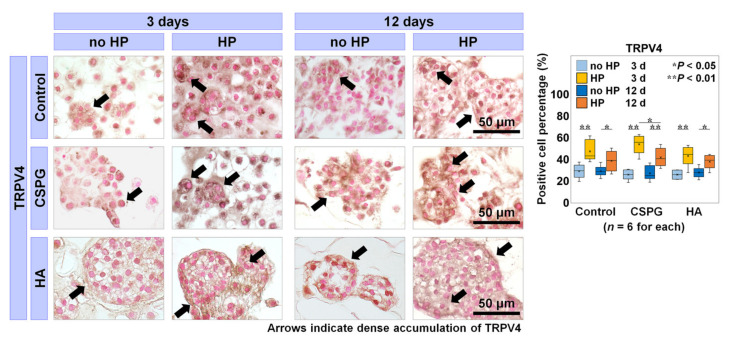
Involvement of transient receptor potential vanilloid-4 (TRPV4) in the effects of augmenting extracellular matrix (ECM) on bovine nucleus pulposus (bNP) cells under hydrostatic pressure (HP). The accumulation of TRPV4 in black counterstained with Contrast Red and positive cell percentage of TRPV4 are shown (*n* = 6 for each). Arrows indicate intense accumulation, each section is 7 μm thick, and the bar indicates 50 μm. Data are presented with box plots. Multi-way repeated measures analysis of variance (ANOVA) with the Bonferroni post hoc test was used. * *p* < 0.05 and ** *p* < 0.01.

Intense TRPV4 staining was found under HP at 3 days with all materials, localized at the surface of bNP cells/clusters; staining was weaker without HP; intense staining was diminished by 12 days. The percentage of cells positive for TRPV4 was significantly higher under HP than no HP with all materials both at 3 and 12 days (control, 3 days, *p* < 0.001; 12 days, *p* = 0.03; CSPG, 3 days, *p <* 0.001; 12 days, *p* = 0.002; HA, 3 days, *p* < 0.001; 12 days, *p* = 0.02). Immunopositivity was lower at 12 days than 3 days under HP with all materials, although this difference reached statistical significance only with CSPG (*p =* 0.02) (Figure 3). These results indicate that the activation of TRPV4 is involved in the cellular responses to dynamic HP, especially at early phases of culture.

## 3. Discussion

### 3.1. Effects of Augmenting ECM on bNP-Cell Metabolism

Several ECM-based materials, such as hyaluronan-based, alginate-based, and collagen-based ones, appear to stimulate IVD-cell metabolism, although their clinical and therapeutic strategies have not been fully addressed [13]. We managed to shed light on the effects of augmenting CSPG and HA on metabolic turnover in bNP-cells under alternating cyclic and constant HP, mimicking diurnal spinal motion. As expected, we found that augmenting ECM specifically with CSPG promoted regenerative turnover in bNP cells through restoring the native ECM microenvironment. The significant *Acan* upregulation and *Col1a1* downregulation with CSPG under no HP at 3 days demonstrates the anabolic and anti-fibrotic effects of CSPG early in incubation. Furthermore, the significant upregulation of *Acan* and *Col2a1* with CSPG under HP at 12 days indicates the synergy of augmenting CSPG with dynamic HP at a later time point. The *Has2* expression and *Acan* expression showed a similar tendency, which is consistent with the work of Holmes [21], Roughley [22], and Sivan [23] reporting the close association between HA and aggrecan turnover both in the articular cartilage and the IVD.

In addition, repetitive changes in HP also elicited catabolic *Mmp13* downregulation and anti-catabolic *Timp2* upregulation in response to slight *Mmp13* upregulation even without any material augmentation. Since disc-ECM catabolism indicated by MMPs is balanced by the inhibitory effects of TIMPs [24], dynamic HP stimulates anti-catabolic turnover of bNP cells and suppresses catabolic changes. In this study, HA inhibited ECM synthesis and strongly promoted catabolic turnover with or without HP. Augmenting HA has an anabolic and anti-inflammatory effect on human chondrocytes [25], and intra-articular administration of HA is an established treatment for such joint diseases as knee osteoarthritis [26]. However, HAS activity appears to be an important modulator, and the anabolic and anti-catabolic effects of extracellular HA without activating HAS could be limited in vitro [27]. Because the augmented HA used in our experiments is commercially available as a cell scaffold for in vitro cell culture, careful interpretation is required in evaluating the clinical relevance of our results.

### 3.2. The Relationship between the Augmented Materials and OP

The HP at physiological range (0.3–1.0 MPa) increases ECM synthesis in bovine, dog, and rabbit NP cells in vitro [28,29,30], while higher HP (>2.5 MPa) induced a catabolic trend, increasing MMP3 activity and decreasing matrix synthesis [31,32]. We developed and applied a repetitive regimen of cyclic HP followed by constant HP at high osmolality to mimic physiological changes in intradiscal pressure and to reproduce the microenvironment in disc homeostasis [11].

The OP is generated by water content absorbed within the tissue [6,7] and influences bNP cell metabolism [10,11]. Therefore, to reproduce the microenvironment in IVD homeostasis, we chose CSPG and HA in this study, both of which are capable of absorbing abundant interstitial fluid. Despite the hydrophilic properties of both materials, our results for bNP-cell anabolism and catabolism were opposite between these materials. Aggrecan, the primary CSPG in the IVD, generally contains approximately 100 chains of chondroitin sulfate and 30 keratan sulfate chains [15,16]. The negatively charged chondroitin sulfate chains contribute to the major function of aggrecan as a structural proteoglycan, holding large amounts of water in the ECM [15]. The high OP, including the capability to swell and resist compressive loads, is generated by the hydrated chondroitin sulfate chains [8,23]; meanwhile, HA has no sulfate chains, which is unique and distinct from other glycosaminoglycans [17,33]. We regard the presence of sulfate chains, generating OP, as the biggest factor in the different responses of the bNP cells between CSPG and HA in the current study. The augmented CSPG provides OP to the bNP cells and stimulates ECM synthesis early in culture, and then newly synthesized ECM would maintain OP and the microenvironment within the semi-permeable membrane pouch during later phases. The semi-confined pouch not only holds both augmented CSPG and synthesized ECM around the cells but also helps the generated OP directly affect the cells. Based on our findings, we consider ECM-associated OP, generated by augmented or synthesized ECM, a key player in NP cell metabolism.

### 3.3. HP Mechanotransduction via TRPV4

The TRPV4 staining was more intense with HP than without HP, both at 3 and 12 days; and it diminished by 12 days under HP although the difference was statistically significant only with CSPG. Thus, TRPV4 activation is implicated in transmitting the effects of HP to the bNP cell metabolism. Simultaneously, ECM was newly synthesized and accumulated around bNP cells over time, as shown by immunohistology against KS. The accumulated ECM likely altered membrane characteristics and subsequently decelerated TRPV4 activation. Although TRPV4-positive cell percentages were not significantly different among the groups, the accumulation of TRPV4 in HA was not as robust as in the control and CSPG, suggesting that the presence of HA might interrupt TRPV4 activation. This is another reason why we regard the physicochemical properties of HA as a possible cause of unfavorable response to dynamic HP, whereas CSPG did not diminish TRPV4 activation. We also hypothesize that dynamic HP stimulates metabolic turnover via TRPV4 activation early in incubation and involves another signal pathway with longer incubation due to ECM accumulation [19,20], which remains to be investigated in future studies.

### 3.4. Limitations

This study has several limitations. First, the rheology of CSPG and HA was not examined, and the concentrations of the materials were preset. Although the concentrations of the materials conformed to the manufacturers’ instructions, concentration and viscoelasticity may affect cellular and metabolic turnover in bNP cells. Before attempting clinical translation, rheological assessment, and optimization of the concentration of each material should be performed. Second, the culture duration was sufficient for the gene expression assay but may need to be extended in order to detect histological differences in accumulation of ECM, especially between the control and CSPG. Third, NP-specific markers, such as Brachyury, Keratin 18, and CD24, have not been evaluated because we focused on ECM synthesis and catabolic turnover in this study. Fourth, to clarify the role of TRPV4 in bNP cell metabolism, TRPV4 suppression and overexpression should be needed. Last of all, experiments with degenerated or inflammation-stimulated NP cells are warranted to clarify the regenerative potential of CSPG or HA; these will be needed to examine using human IVD samples.

## 4. Materials and Methods

### 4.1. Antibodies and Reagents

All antibodies and reagents used are listed in Table S1.

### 4.2. Isolation and Pre-Culture of bNP Cells

Bovine tails (from cows 2–3 years old) were purchased from a local slaughterhouse certified by United States Department of Agriculture (USDA), and caudal NP tissues were harvested. The cows are skeletally mature, and their NP-cell phenotypes are relevant to human NP cells [34]. Five tails were used for one set of experiments, which was repeated six times (*n* = 6). The harvested bNP tissues were digested in 0.10% collagenase (CLS-1), dissolved in Ham’s F12 nutrient mixture (F-12), and supplemented with 100-units/mL penicillin and 100 µg/mL streptomycin at 37 °C overnight. Then bNP cells were collected, rinsed with Dulbecco’s phosphate-buffered saline (D-PBS), seeded onto 1.5% cell-culture-grade agarose-coated 6-well plates, and incubated in Dulbecco’s Modified Eagle Medium (DMEM)/F-12 (1:1) supplemented with 10% fetal bovine serum (FBS) and 100-units/mL penicillin/100 µg/mL streptomycin at 37 °C, 5% CO_2_ for 2–3 days. After this preincubation, bNP cells/clusters were collected with a pipette under a dissection microscope.

### 4.3. Cell Seeding

**Figure 4:**
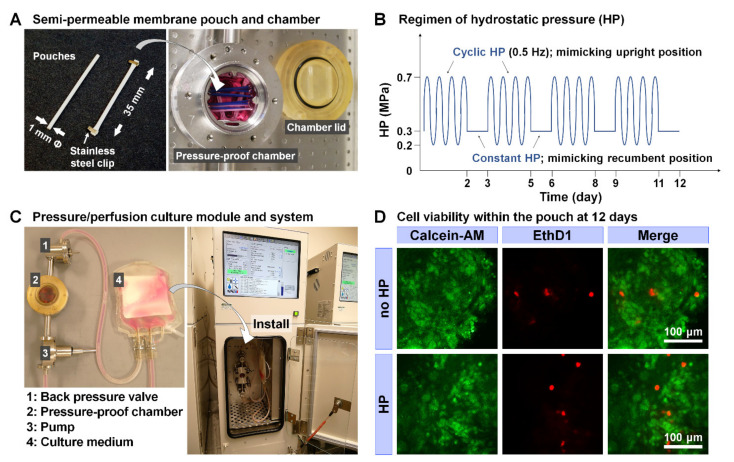
Our semi-permeable membrane pouch culture module and pressure/perfusion culture system. (**A**) Semi-permeable membrane pouches and a pressure-proof chamber. (**B**) Repetitive regimen of 2-day cyclic followed by 1-day constant hydrostatic pressure (HP). (**C**) Macroscopic appearance of pressure/perfusion culture module and system. (**D**) Validation of bovine nucleus pulposus cell viability at 12 days. Calcein-AM indicates live cells and ethidium homodimer-1 (EthD1) indicates dead cells. The bar indicates 100 μm.

For the bNP tissue model, semipermeable membrane pouches were prepared as previously described [11]. Briefly, hollow fiber tubing (1 mm in diameter, polyvinylidene fluoride, 500 kD molecular weight cut-off) was cut into 35 mm lengths, immersed in ethyl alcohol (200 proof) for 30 min, and autoclaved in D-PBS at 121 °C for 15 min. After carefully collecting the bNP cells from each well in the 6-well plate, 1.0 × 10^5^ bNP cells (the DNA equivalent) with D-PBS as a no-material control, with 2 mg/mL CSPG (aggrecan from bovine articular cartilage), or with 10 mg/mL HA-based uncross-linked hydrogel (HyStem^TM^), were injected into the pieces of tubing aseptically using a 200 μL pipette, and both ends of the tubing were closed with stainless steel clips (Figure 4A). The concentrations of CSPG and HA were determined according to the manufacturers’ instructions.

### 4.4. Culture Regimen of HP and Medium Osmolality/Incubation Conditions

The pouches were incubated under two different sets of culture conditions based on our previous study [10,11]: (1) no HP, in which pouches were placed in culture medium under atmospheric pressure; and (2) HP, in which 2-day cyclic HP (0.2–0.7 MPa, 0.5 Hz) followed by 1-day constant HP (0.3 MPa) was repeated 4 times over 12 days (Figure 4B). Both sets of conditions also included 37 °C, 5% CO_2_, and 3% O_2_ to stimulate a physiologic hypoxic IVD environment [3,4]. High-osmolality medium at 450 mOsm/kg H_2_O was made using 4.6-g/L sodium chloride and the osmolality was confirmed with a freeze-point osmometer (MICRO-OSMETTE^TM^). Under no HP, the pouches were suspended within a stainless-steel mesh basket held in 100 mL medium with a stirrer to maintain sufficient mass transfer through the pouches. Under HP, the pouches were placed in a culture chamber filled with the medium to load cyclic or constant HP with medium replenishment at 0.1 mL/min using a pressure/perfusion culture system (TEP-2; Figure 4C). At 12 days, cell viability of the bNP cells/clusters within the pouches was validated with calcein-AM for live cells and ethidium homodimer-1 (EthD1) for dead cells (LIVE/DEAD^TM^ Viability/Cytotoxicity Kit) according to the manufacturer’s instruction, which demonstrated predominant population of live cells (Figure 4D).

### 4.5. RNA Isolation and Real-Time Reverse Transcription–Polymerase Chain Reaction (RT–PCR)

The bNP cells/clusters were harvested at 3 and 12 days. Total RNA was extracted using the RNeasy^®^ mini kit, and 0.3 μg RNA was reverse-transcribed with random primers (High-Capacity cDNA Reverse Transcription Kit). Messenger RNA (mRNA) expression levels of *Acan*, *Col1a1*, *Col2a1*, *Has2*, *Mmp13*, and *Timp2* relative to *glyceraldehyde 3-phosphate dehydrogenase* (*Gapdh*) as an endogenous control were evaluated in quadruplicate by real-time RT–PCR using TaqMan^TM^ gene expression master mix and fluorescent-labeled specific primers. The commercially available validated primers used were as follows: *Acan*, Bt03212189_m1; *Col1a1*, Bt03225358_g1; *Col2a1*, Bt03251837_mH; *Has2*, Bt03212694_g1; *Mmp13*, Bt03214051_m1; *Timp2*, Bt03231007_m1; *Gapdh*, Bt03210919_g1 (TaqMan^TM^ probes). Measurements were performed using the QuantStudio 5 Real-Time PCR System. Relative mRNA expression was analyzed by the 2^−ΔΔCt^ method using ExpressionSuite Software v1.0.4 [35]. The value of the no-material control sample with D-PBS under no HP at 3 days was set at 1.0.

### 4.6. Immunohistology

At 3 and 12 days, the bNP cells/clusters were fixed with 2% paraformaldehyde/0.1 M cacodylate buffer (pH 7.4) at 4 °C, embedded in paraffin, and cut into 7 μm sections. Dewaxed sections were incubated with primary antibodies against KS (1:500), MMP13 (1:50), and TRPV4 (1:100) overnight. The sections were then rinsed and incubated with a biotinylated secondary antibody (VECTASTAIN^®^ Elite ABC-HRP kit) for 30 min. The color was developed with 3,3′-diaminobenzidine and nickel (DAB substrate kit). Counterstaining was performed with Harris’s hematoxylin for KS, and with Contrast Red for MMP13 and TRPV4. The number of positive cells was counted in four random high-power fields (×400) using the ImageJ software (https://imagej.nih.gov/ij/, accessed on 4 October 2018). The positive cell percentage for MMP13 and TRPV4 was calculated relative to the total number of hematoxylin- or Contrast Red-positive cells.

### 4.7. Statistical Analysis

Data are expressed as box plots in the graphs. Multi-way repeated measures analysis of variance (ANOVA) with the Bonferroni post hoc test was used in real-time RT–PCR (relative values to the no-material control sample under no HP at 3 days) and immunohistology. Pearson correlation analysis was performed to assess the correlation between *Mmp13* and *Timp2* expression at 12 days. The *p*-values < 0.05 were regarded as statistically significant using IBM SPSS Statistics 23.0 (IBM, Armonk, NY, USA).

## 5. Conclusions

We evaluated metabolic turnover in isolated bNP cells with augmented CSPG and HA under a repetitive regimen of cyclic and constant HP mimicking the circadian HP changes within IVD. Augmenting CSPG elicited anabolic *Acan* enhancement and fibrotic *Col1a1* suppression at early time point in culture. In addition, CSPG was suggested to have anabolic synergy with dynamic HP on ECM synthesis at later time points. Our repetitive regimen of dynamic HP also elicited catabolic *Mmp13* downregulation. However, HA augmentation did not provide anabolic stimulation, but promotes catabolic *Mmp13* expression regardless of the presence of HP, which demonstrated that HA was not as beneficial as CSPG in our culture model. The TRPV4 may be involved in HP signal transduction, especially early in incubation. The CSPG has the potential to facilitate IVD regeneration by reproducing the ECM microenvironment around the bNP cells.

## Data Availability

The data presented in this study are available from the corresponding author on reasonable request.

## Supplementary Materials

The following is available online at https://www.mdpi.com/article/10.3390/ijms22116015/s1, Table S1: List of the antibodies, reagents, and instruments used.
