# Supplementary material for: Augmented Chondroitin Sulfate Proteoglycan Has Therapeutic Potential for Intervertebral Disc Degeneration by Stimulating Anabolic Turnover in Bovine Nucleus Pulposus Cells under Changes in Hydrostatic Pressure
Source: Int J Mol Sci. 2021 Jun 2;22(11):6015. doi: 10.3390/ijms22116015 (PMC8199579; doi:10.3390/ijms22116015)
Supplement: Supplementary file 1 [file ijms-22-06015-s001.zip › ijms-1184804-supplementary.pdf]

Table S1. List of the antibodies, reagents, and instruments used.

Product	Catalog number	Manufacturer
Antibody		
Keratan sulfate (mouse, monoclonal)	sc-73518	Santa Cruz Biotechnology (Santa Cruz, CA)
MMP13 (rabbit, polyclonal)	LS-B3168	LifeSpan BioSciences, Inc. (Seattle, WA)
TRPV4 (rabbit polyclonal)	LS-A8583	LifeSpan BioSciences, Inc. (Seattle, WA)
Cell culture		
DMEM	11965092	Gibco (Waltham, MA)
Ham's F12	11765054	Gibco (Waltham, MA)
FBS	10437028	Gibco (Waltham, MA)
Penicillin/streptomycin	15140122	Gibco (Waltham, MA)
ITS	41400045	Gibco (Waltham, MA)
D-PBS	14190144	Gibco (Waltham, MA)
Collagenase type 1	LS004196	Worthington Biochemical (Lakewood, NJ)
Hollow fiber tubing	S9320101, S9320102	Repligen (Waltham, MA)
Pure ethyl alcohol	459836	Sigma-Aldrich (St. Louis, MO)
MICRO-OSMETTE™ osmometer	5004	Precision Systems, Inc. (Natick, MA)
Pressure/perfusion culture system		Purpose (Shizuoka, Japan)
Aggrecan from bovine articular cartilage	A1960	Sigma-Aldrich (St. Louis, MO)
HyStem® Cell culture scaffold kit	HYS020	Sigma-Aldrich (St. Louis, MO)
Staining		
VECTASTAIN® Elite ABC-HRP kit	PK6200	Vector Laboratory (Burlingame, CA)
DAB substrate kit	SK4100	Vector Laboratory (Burlingame, CA)
Harris's hematoxylin	HHS16	Sigma-Aldrich (St. Louis, MO)
Contrast Red	5540-0001	Sera care (Milford, MA)
Real-time RT–PCR		
RNeasy mini kit	74104	Qiagen (Hilden, Germany)
High-Capacity cDNA Reverse Transcription Kit	4368814	Applied Biosystems (Foster City, CA)
TaqMan™ gene expression master mix	4369016	Applied Biosystems (Foster City, CA)
Pre-designed PCR primers		Life Technologies (Carlsbad, CA)
QuantStudio 5 Real-Time PCR System	A28140	Applied Biosystems (Foster City, CA)

DAB = 3,3'-diaminobenzidine; DMEM = Dulbecco's modified Eagle's medium; D-PBS = Dulbecco's phosphate-buffered saline; FBS = fetal bovine serum; ITS = insulin-transferrin-selenium; MMP13 = matrix metalloproteinase 13; RT–PCR = reverse transcription–polymerase chain reaction; TRPV4 = transient receptor potential vanilloid-4.
